# Foster Kennedy syndrome caused by tuberculous brain abscess: A case report

**DOI:** 10.1016/j.jctube.2021.100265

**Published:** 2021-07-22

**Authors:** Rizaldy Taslim Pinzon, Vanessa Veronica

**Affiliations:** a Duta Wacana Christian University School of Medicine, Yogyakarta, Indonesia; b Bethesda Hospital, Yogyakarta, Indonesia

**Keywords:** Tuberculous brain abscess, Foster Kennedy syndrome, Central nervous system tuberculosis

## Abstract

**Background:**

A tuberculous brain abscess is an unusual form of central nervous system tuberculosis, whereas Foster Kennedy syndrome is a rare condition characterized by ipsilateral optic atrophy, contralateral papilloedema, and ipsilateral anosmia. Foster Kennedy Syndrome and tuberculous brain abscess both are rare conditions with limited study. We report the first case of Foster Kennedy syndrome associated with tuberculous brain abscess.

**Case presentation:**

A 32 years old male presented with severe headache, memory impairment, speech difficulty, a slight right-side weakness, and vision impairment for eight weeks. The symptoms began with a low-grade fever, a dry cough, and a loss of appetite, which intensified over time. The patient was generally wasted and drowsy. Physical examination showed right-sided hemiparesis. An enhanced lesion in the left frontal lobe was seen on a plain computed tomography scan accompanied by perifocal edema. An open craniectomy was performed, and antituberculous therapy was begun immediately. The disability had significantly improved in a month.

**Conclusion:**

In patients with Foster Kennedy syndrome, a tuberculous brain abscess should be considered. Patients who have been diagnosed must undergo surgical surgery as well as antituberculous therapy to recover fully.

## 1 Introduction

Foster Kennedy syndrome is a rare condition that is believed to involve 1% to 2.5% of intracranial masses [1]. One of the intracranial masses that can cause Foster Kennedy Syndrome is the frontal lobe abscess [2].

Tuberculosis is the most widespread and persistent human infectious disease in the world. Tuberculosis infection can involve many organs and resemble many other diseases. Tuberculosis generally affects the lungs (pulmonary tuberculosis) but can affect other organs (extrapulmonary tuberculosis) [3].

Tuberculous brain abscess (TBA) is a rare but severe condition. Tuberculous brain abscess (TBA) is one of the uncommon types of central nervous system tuberculosis. Even in countries where tuberculosis is a troubling public health concern – Indonesia is one of the eight countries with the highest tuberculosis rate in 2019 -it is rarely reported [3], [4].

Despite the increases in the incidence of tuberculosis in developed countries, the lack of published cases of tuberculous brain abscess and Foster Kennedy syndrome is of great interest.

## 2 Case presentation

A 32 years old male presented to our hospital's emergency department with severe headache, memory impairment, speech difficulty, slight right-sided weakness, and vision impairment. The complaint began eight weeks before hospital admission and worsened two weeks before admission. The symptoms began with a low-grade fever, a dry cough, and a loss of appetite, which intensified over time. The patient visited primary care physicians and the district hospital without improvement, and the chest radiograph result was inconclusive.

The patient was first referred to our hospital's emergency room and then to our neurology department. The emergency room physician noted mild spastic right-side weakness without any other abnormality on physical examination, chest radiography, and routine laboratory examinations. The patient also complained of weight loss and night sweating when he was admitted.

On physical examination, the patient was generally wasted and drowsy; he was oriented but sleepy. He had a surface temperature of 38 °C, pulse rate of 90 beats/minute, respiratory rate of 16/minute, and blood pressure of 110/80 mmHg; neck stiffness was absent. An examination of the lungs, heart, and abdomen indicated no apparent anomalies. The neurology examination showed a right-sided hemiparesis, with the proximal and distal strength of 4 out of 5 being detected, and a bilateral Babinski sign was present. The results of every cranial nerve and sensory examination were normal. The patient was consulted to the ophthalmology department for further examination due to a vision impairment complaint. The result was unilateral visual loss with fundoscopy findings of compressive optic atrophy in one eye and contralateral papilledema due to increased intracranial pressure.

**Fig. 1:**
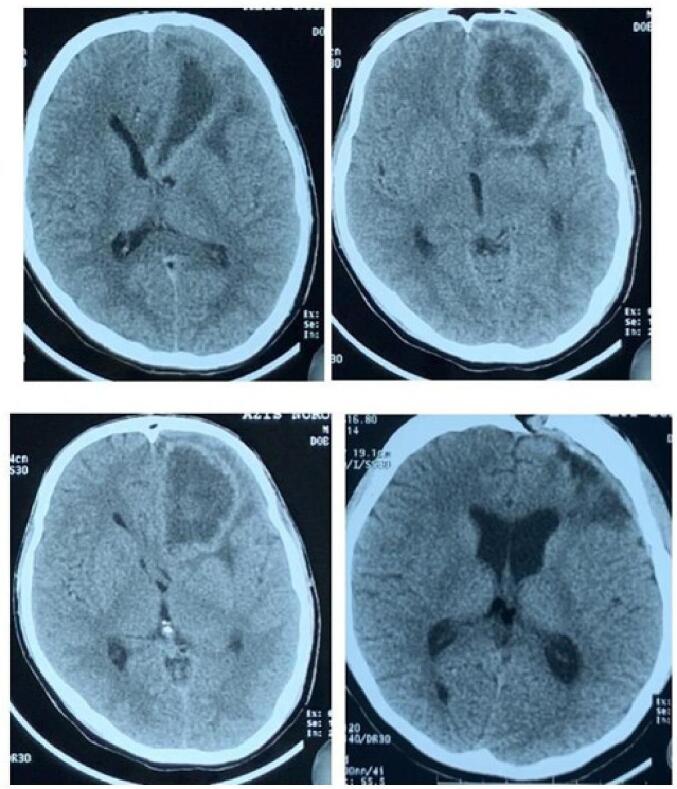
An enhanced lesion in the left frontal lobe on CT-scan.

A computed tomography (CT) scan of the head showed an enhanced lesion in the left frontal lobe, surrounded by perifocal edema (Fig. 1). The patient's sputum acid-fast bacilli laboratory test was positive.

The abscess was removed by an open craniectomy, and antituberculous therapy was begun immediately. Isoniazid (225 mg), rifampicin (450 mg), ethambutol (825 mg), and pyrazinamide (1200 mg) comprised the antituberculous treatment regimen. Vitamin B6 was also applied to the medications, as mentioned earlier. Within a few days, the patient's drowsiness subsided, and his overall health improved after two weeks, but he still had a fever. In about a month, the disability had significantly improved.

## 3 Discussion

Foster Kennedy syndrome is a rare condition with the triad of ipsilateral optic atrophy, papilloedema in the contralateral eye, and anosmia ipsilaterally [5], [6]. Robert Foster Kennedy, who discovered the triad in 6 patients, first reported this condition in 1911 [6]. Its etiology remains unclear, but it is hypothesized that the syndrome is due to compression of the optic nerve, compression of the olfactory nerve, and elevated intracranial pressure due to mass compression [7]. Apart from the triad of Foster Kennedy syndrome, other manifestations of this syndrome include headache, weakness, emotional lability, cognitive impairment, nausea, and vomiting [8].

Tumors are the leading cause of Foster Kennedy syndrome. However, non-neoplastic conditions, one of which is frontal lobe abscess, may also contribute to [2]. Other intracranial masses reported causing Foster Kennedy syndrome are craniopharyngioma, pituitary adenoma, plasmacytoma, nasopharyngeal angiofibroma, neuroblastoma, and aneurysm [9].

Tuberculous brain abscess – one of the uncommon types of central nervous system tuberculosis – typically comes from a focus of infection outside the brain, such as the lungs, spread hematogenously to the brain**.** A tubercle focus on the brain can evolve to tuberculoma or abscess when there is a significant inoculation size and poor cell-mediated immunity – that is why tuberculous brain abscesses are more common in immunocompromised patients than in immunocompetent patients [10].

The gold standard for tuberculous brain abscess diagnosis is culture, but it does not have a high sensitivity (below 70%). A large, single, hypodense lesion with edema can commonly be seen on a non-contrast CT scan of tuberculous brain abscess. Contrast CT scan shows a thin ring enhancement; however, it may evolve into a thick ring, thus distinguish it from pyogenic abscess [11]. In contrast to tuberculoma, histopathological examinations on tuberculous brain abscess do not show both granulomatous inflammation or Langhans giant cells. In addition, tuberculous brain abscess has bacterial abscess-like vascular granulation tissue with necrotic interior wall and fibrous external wall [12]. Chest radiograph does not have a significant role in establishing the diagnosis of tuberculous brain abscess. Eleven cases of infratentorial tuberculoma published in 2013 have been identified, with most cases displaying normal chest radiograph; only one subject having pulmonary tuberculosis [13].

Adults appear to develop supratentorial tuberculoma, whereas children are more likely to have infratentorial tuberculoma [13]. The supratentorial location of the abscess most commonly occurs in the frontal lobe [12]. As mentioned earlier, frontal lobe abscess is one of the non-neoplastic causes of Foster Kennedy syndrome; hence, a tuberculous brain abscess can cause Foster Kennedy syndrome.

Management and prognosis of Foster Kennedy syndrome depend on the underlying condition, in this case, tuberculous brain abscess. Unfortunately, because of the rarity of these disorders, proven guidelines for tuberculous brain abscesses are not available. However, surgical excision integrated with antituberculous therapy (ATT) for 12–18 months was suggested to recover tuberculous brain abscess fully [12].

## 4 Conclusion

Tuberculous brain abscess must be considered in patients with Foster Kennedy syndrome. The diagnosis suspicion can be directed by careful clinical and laboratory assessment. Once diagnosed, patients need to undergo surgical treatment along with antituberculous therapy to achieve full recovery.

## 5 Consent for publication

The patient's written consent was obtained for the publication of this case report by the authors.

## Authors contribution

RTP was responsible for the initial manuscript, data processing, imaging report, and supervision. VV was involved in the manuscript's preparation and editing.

## Funding

This research did not receive any specific grant from funding agencies in the public, commercial, or not-for-profit sectors.

## Declaration of Competing Interest

The authors declare that they have no known competing financial interests or personal relationships that could have appeared to influence the work reported in this paper.
